# Copy number variation and genetic diversity of MHC Class IIb alleles in an alien population of *Xenopus laevis*

**DOI:** 10.1007/s00251-015-0860-3

**Published:** 2015-09-02

**Authors:** Barbara K. Mable, Elizabeth Kilbride, Mark E. Viney, Richard C. Tinsley

**Affiliations:** Institute of Biodiversity, Animal Health & Comparative Medicine, College of Medical, Veterinary & Life Sciences, University of Glasgow, Glasgow, G12 8QQ UK; School of Biological Sciences, University of Bristol, Bristol, BS8 1TQ UK

**Keywords:** *Xenopus laevis*, Introduced species, Polyploidy, MHC, Bottlenecks, Genetic diversity

## Abstract

*Xenopus laevis* (the African clawed frog), which originated through hybridisation and whole genome duplication, has been used as a model for genetics and development for many years, but surprisingly little is known about immune gene variation in natural populations. The purpose of this study was to use an isolated population of *X. laevis* that was introduced to Wales, UK in the past 50 years to investigate how variation at the MHC compares to that at other loci, following a severe population bottleneck. Among 18 individuals, we found nine alleles based on exon 2 sequences of the Class IIb region (which includes the peptide binding region). Individuals carried from one to three of the loci identified from previous laboratory studies. Genetic variation was an order of magnitude higher at the MHC compared with three single-copy nuclear genes, but all loci showed high levels of heterozygosity and nucleotide diversity and there was not an excess of homozygosity or decrease in diversity over time that would suggest extensive inbreeding in the introduced population. Tajima’s *D* was positive for all loci, which is consistent with a bottleneck. Moreover, comparison with published sequences identified the source of the introduced population as the Western Cape region of South Africa, where most commercial suppliers have obtained their stocks. These factors suggest that despite founding by potentially already inbred individuals, the alien population in Wales has maintained substantial genetic variation at both adaptively important and neutral genes.

**Electronic supplementary material:**

The online version of this article (doi:10.1007/s00251-015-0860-3) contains supplementary material, which is available to authorized users.

## Introduction

A major concern for conservation is how changing climates and human-mediated habitat alterations affect genetic diversity in increasingly fragmented and isolated populations. Although alien species are often viewed negatively, in cases where the introduced population is not able to invade a wide proportion of the new habitat, they can make useful models for investigating the genetic consequences of population fragmentation arising from introduction.

*Xenopus laevis,* the African clawed frog, is native to sub-Saharan Africa but has been introduced to multiple locations worldwide. This species was used for human pregnancy tests until the 1970s, as well as a model for developmental biology and genetics (Gurdon 1996). Alien *X. laevis* populations are established in many parts of the world, attributed to release of individuals originating from the southwest region of the Western Cape Province of South Africa (Lillo et al. 2013; Lillo et al. 2011; Measey et al. 2012; Tinsley and Kobel 1996), which has a Mediterranean climate. Populations have become established in various regions with a temperate climate, including the UK. In Wales, an alien, but closed, breeding population of *X. laevis* was founded about 50 years ago, prior to its extinction in 2010 (Tinsley et al. 2015b). Mark-recapture data were collected from this population for 30 years, with records of birth dates, gender, population size and infections with the monogenean *Protopolystoma xenopodis* (Tinsley et al. 2012). This population therefore provides an interesting model to examine how adaptation to a novel environment can impact genetic diversity of a fragmented population that was likely founded by a small number of individuals.

A complicating but intriguing aspect of *X. laevis* biology is that it is a tetraploid, meaning that it arose through genome duplication, possibly following a hybridisation event (allopolyploidisation). The whole genome duplication (WGD) event is thought to have occurred 29–66 mya (Chain and Evans 2006), and there has subsequently been substantial loss of duplicate gene expression, although many genes are retained in duplicate (Chain et al. 2011; Hellsten et al. 2007; Morin et al. 2006; Semon and Wolfe 2008). Differential loss or retention of duplicate genes could alter the consequences of genetic bottlenecks, or the ability to expand into new environments. In plants, polyploidy has been associated with invasiveness (defined as the ability to become established in new environments) due to the increased genomic flexibility that is predicted to accompany WGD (Ainouche et al. 2009; Pandit et al. 2011).

It has been proposed that *X. laevis* has played a role in the spread of important amphibian diseases such as the chytrid fungus (Weldon et al. 2004) and ranavirus (Robert et al. 2007), even though individuals do not normally present disease symptoms. One possibility for this is that duplication of genes coding for the immune machinery (or differential loss of duplicate copies) has conferred increased tolerance and/or resistance to pathogens and parasites (Jackson and Tinsley 2001; Jackson and Tinsley 2003). Even in diploids, duplication and divergence of genes coding for aspects of immune function is thought to have been important for generating redundant copies that can be recruited for tissue- or pathogen-specific defence, but also for enabling rapid expansion of the available repertoire through nonreciprocal recombination among copies (i.e. gene conversion; Martinsohn et al. 1999; Ohta 1991; Spurgin et al. 2011; van Oosterhout 2009). This has resulted in extensive copy number variation (CNV) between species, within species and even within populations of diploid organisms (Llaurens et al. 2012). While tandem duplications certainly have contributed, the ‘big bang’ theory suggests that historic multiple rounds of WGD have resulted in the most dramatic changes in copy number of immune genes (reviewed by Flajnik and Kasahara 2010). Polyploids could thus be more resistant to parasites compared to their diploid progenitors because of greater allelic diversity of molecules of the immune system (Jackson and Tinsley 2001, 2003; King et al. 2012). In addition, the process of hybridisation could expand immune gene capacity through potentially advantageous interactions between genes encoded by each subgenome (Comai et al. 2000; Evans 2007). Alternatively, the development of new polyploids could substantially perturbate the immune repertoire, possibly leading to immunopathology, including due to autoimmunity (Zou and Secombes 2011). In plants, for instance, polyploids are sometimes, but not always, more resistant to attack by parasites than their diploid progenitors (Nuismer and Thompson 2001; Schoen et al. 1991; Thompson et al. 2004). An intriguing possibility is that this might depend on whether immune genes are retained in duplicate or dynamic loss of duplicate copies within or between populations, but this has not been investigated.

Despite being one of best studied ectothermic vertebrate models of immune function (e.g. Chida et al. 2011; Maniero et al. 2006; Robert and Cohen 2011; Robert and Ohta 2009), surprisingly little is known about natural variation in immune genes in *X. laevis*, or how immune gene variation is impacted by population bottlenecks in nature or inbreeding in laboratory colonies. This is reflected in lack of a standardised nomenclature for MHC genes in *Xenopus*. Some tetraploid (*X. laevis*) and octoploid (*Xenopus vestitus*) species express the same number of Major Histocompatibility Complex (MHC) copies as a closely related diploid species (*Xenopus tropicalis*), suggesting loss of copy number. In contrast, a more recently diverged dodecaploid species (*Xenopus ruwenzoriensis*) shows expression of additional copies, which suggests retention of duplicates (Du Pasquier and Blomberg 1982; Flajnik 1996; Kobari et al. 1995; Kobel and Du Pasquier 1986; Sammut et al. 2002; Sato et al. 1993; Shum et al. 1993). The MHC Class IIb region of *X. laevis* is organised into three loci, none of which appears to be retained in duplicate (Kobari et al. 1995). There are no published data available on variation at the MHC from multiple individuals sampled from a wild population of *X. laevis*, so it is not known whether MHC copy number is a fixed or variable trait. Published sequences also do not include information on heterozygosity of individuals because the focus has been on characterising the structure of the MHC based on partially inbred laboratory-reared animals (Klein et al. 2002; Kobari et al. 1995; Sato et al. 1993).

However, there has been comparison of variation both in mitochondrial (Evans et al. 1997) and single-copy nuclear genes (Bewick et al. 2011; Furman et al. 2015) for multiple individuals sampled from discrete populations in the native range of *X. laevis* in Africa, including the Western Cape Province of South Africa. Sequencing the same sets of genes in the alien Welsh population would allow an assessment of relative diversity at the MHC compared to other nuclear genes and to identify the likely source of the introduced population.

The overall purpose of this study was to use the Welsh *X. laevis* population as a model to investigate the impacts on genetic diversity of reproductive isolation in a novel alien habitat. We addressed four specific questions: (1) Is there evidence that the Class IIb region of the MHC is under diversifying selection? (2) Is there evidence for copy number variation at the MHC? (3) How much diversity and heterozygosity are maintained at the MHC compared to single-copy nuclear genes? (4) What was the likely source of the introduced population?

## Materials and methods

### Samples, DNA extractions and PCR conditions

The individuals used in this study were part of the long-term mark-recapture study described above (Additional file: Table S1; Tinsley et al. 2015a, b). Twelve of the 18 individuals sampled were born in 1993 (about 30 years after the initial population establishment), with the remainder born between 1999 and 2005; this sampling thus provides some perspective on changes in the population over time.

For the MHC, we targeted exon 2 of the Class IIb region using conserved primers described by Hauswaldt et al. (2007) that were designed for *Rana temporaria* but based on *Xenopus* spp. sequences (Haus-MHC-F: CCS CAG AKG ATT WCG TGW MTC A; Haus-MHC-5R: TGT CTG CAG ACT GTY TCC ACC HCA GCC). This exon contains most of the β1 domain, which contains the peptide-binding region (PBR; Kobari et al. 1995). For comparison with published sequences from the native range in South Africa, we sequenced the mitochondrial 16S rDNA using the primers *16Sc* (GTRGGCCTAAAAGCAGCCAC) and 16Sd (CTCCGGTCTGAACTCAGATCACGTAG) described in Moriarty and Cannatella (2004). We also sequenced three single-copy nuclear genes, using primers described in Bewick et al. (2011): two housekeeping genes (*Prmt6*, protein arginine methyltransferase; Xlaexon4_for1: GAC CRS GAG TAT TTC CAG TGC TAC TC; Xlaexon4_rev1: CAT AYG GCG ACG TMG ATA AAG TGA C and *Mogs*, mannosyl-oligosaccharide glucosidase; Xlaexon5_for2: CTG AAG ATG AGC GGC ATG TGG ATC TG; Xlaexon5_rev2: CTT CAG CCA TGA TTA GTA CCA C) and an immune - function - related coding gene not at the MHC (*Rag2*, recombination activating geneXla-Rag2_for_45: CTGGGAGTAATACATCATGATC; Xla-Rag2_rev_1149: CCTCGTCAAAATGTTCCCGTCTCTG). The three nuclear genes were chosen because: (1) they have been shown to be single copy in *X. laevis* and do not contain length variants, enabling resolution of heterozygous positions based on direct sequencing; (2) they were found to amplify reliably across all samples in initial tests; and (3) sequences were available in GenBank that included IUPAC ambiguity codes, enabling resolution of complete genotypes for heterozygous individuals and comparison with sequences from a broader geographic sampling (Furman et al. 2015).

Polymerase chain reactions were run using a standard set of conditions with Invitrogen *Taq* DNA polymerase (Invitrogen Inc., Paisley, UK) and its associated reagents, with annealing temperatures optimised for each set of primers (58 °C for MHC, 53 °C mtDNA, and 60 °C for other nuclear genes). Reactions were run in 20 μl final volume using 2 μl of 10× PCR buffer, 1 μl of 50 mM MgCl_2_, 2 μl 2 mM dNTPs (Promega Inc., Southampton, UK), 0.2 μl of 500 U/μl Taq and 0.2 μl 10 mM stocks of each primer. Cycling conditions were 94 °C for 3 min, annealing for 1 min, 72 °C for 2 min, followed by 35 cycles of 94 °C for 30 s, annealing for 30 s, 72 °C for 2 min, with a final extension at 72 °C for 6 min.

For the MHC, amplicons were cloned into plasmid vectors using Invitrogen TOPO TA cloning kits for sequencing (Invitrogen Inc., Paisley, UK). A target of at least 18 clones per individual was set for sequencing of plasmids, purified using QIAprep Miniprep Kits (Qiagen Inc, Manchester, UK). Clones were bidirectionally sequenced by the GenePool, Edinburgh, using the Universal vector primers M13F and M13R. Chromatographs were assembled into contigs and base-calling errors corrected using Sequencher 4.0 (Gene Codes Corp, Ann Arbor, MI). Consensus sequences of sets of sequence variants within an individual were used to resolve alleles (referred to from this point on as haplotypes). In cases where a sequence variant was found in a single clone within an individual, this was only considered as a haplotype if also found in at least one other individual. The number of Class IIb haplotypes resolved per individual was plotted against the number of clones sequenced to determine whether it was likely that sequence variants might have missed (Additional file: Fig. S1).

For the other genes, amplification products were purified with USB Exosap-IT cleanup kits (Affymetrix Inc., USA) and sent to the GenePool in Edinburgh for direct sequencing in both directions. For the nuclear genes, haplotypes were resolved by eye, using the principle that the most common haplotype in a population was the basal sequence.

### Selection at the MHC

**Fig. 1:**
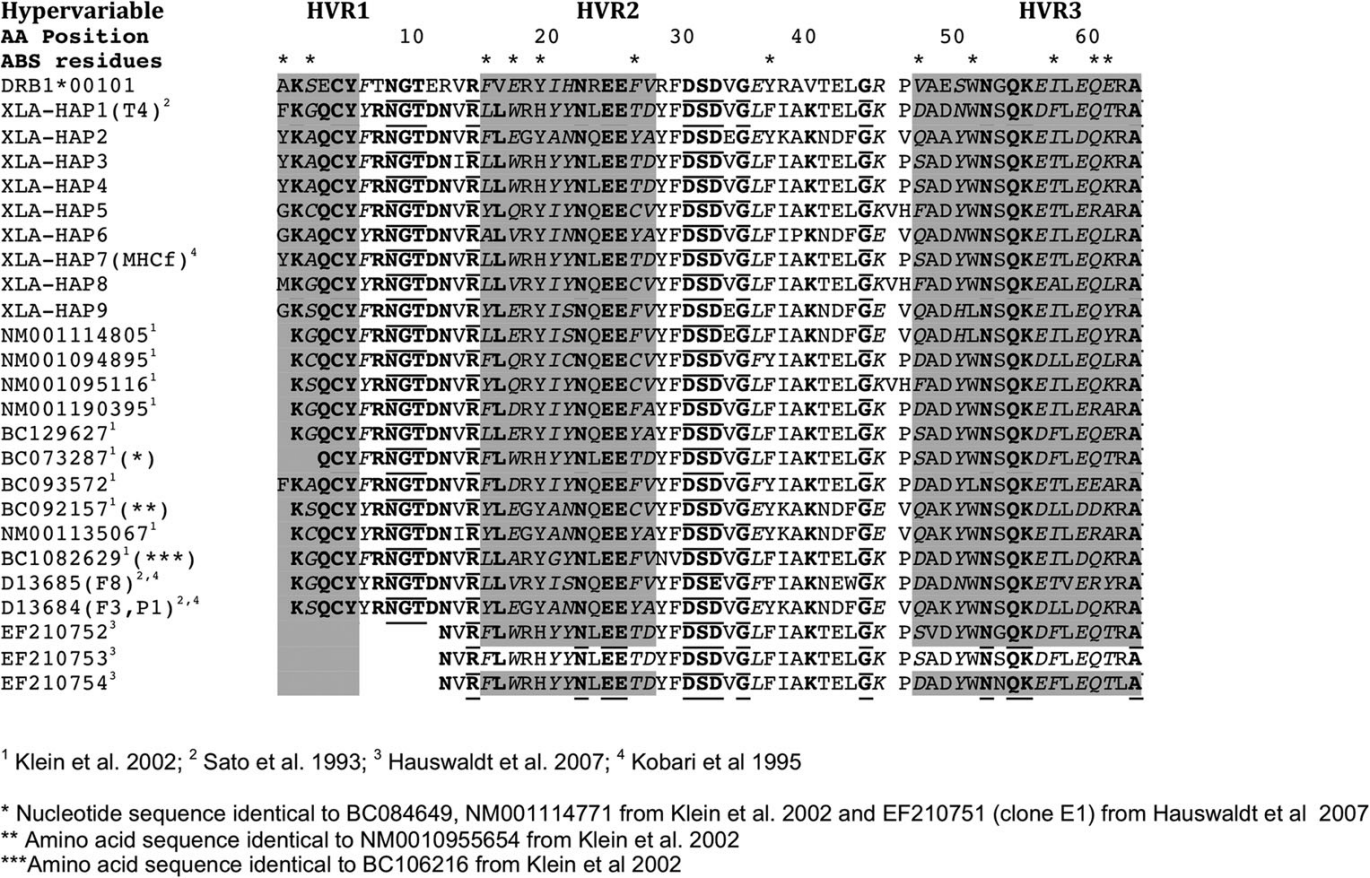
Comparison of MHC Class IIb sequences from Welsh *X. laevis* (XLA-HAP), published laboratory *X. laevis* (accession number indicated) and a domestic dog sequence from the DRB1 locus (DRB1*00101). Hypervariable regions (HVR; *shaded*) and antigen binding sites (ABS, *asterisks*) are as defined in Marsden et al. (2009) for canid haplotypes based on human HLA sequences described in Bondinas et al. (2007). Amino acid numbers start from the beginning of the exon 2 region sequenced for Welsh *Xenopus. Bold* indicates amino acids conserved among all *Xenopus* haplotypes and *underlined* are those also conserved across highly divergent canid species (domestic dogs and African Wild Dogs; taken from Marsden et al. 2009). In total, 21/64 amino acids were conserved among dogs and all *Xenopus*, with one occurring in an HVR but none at predicted ABS. Residues found to be under positive selection using the REL method, as implemented in datamonkey, are indicated by *italics*; the strongest selection was found at sites 3, 16 and 62, corresponding to one ABS in each of the HVR. Positive selection was detected at these sites for all other methods used as well (IFEL, FEL, MEME, SLAC). REL was the least conservative test, but sites 48 and 58 were also identified by most other methods; these are also at predicted ABS sites

BLAST was used to identify all other *X. laevis* sequences available in GenBank (Hauswaldt et al. 2007; Klein et al. 2002; Kobari et al. 1995; Sato et al. 1993), which were downloaded and aligned to the Welsh sequence variants (Fig. 1). All available sequences for MHC loci were from at least partially inbred laboratory-reared individuals (which we will refer to jointly as “laboratory colonies”). The translated amino acid sequence was compared to an amino acid alignment from Marsden et al. (2009) that compared variation in highly endangered African wild dogs to domestic dogs, as an indication of how sequence conservation in the MHC of the alien *X. laevis* population compared to other fragmented populations. Hypervariable regions (HVR) and antigen binding sites (ABS) were predicted based on characterisation of human HLA sequences (Bondinas et al. 2007).

To test for selection acting on Class IIB alleles in relation to HVR and ABS predicted for humans, the datamonkey server (www.datamonkey.org), which implements statistical tests associated with the programme HyPhy (Pond et al. 2005), was used to analyse an alignment of all unique haplotypes in the Welsh populations with the laboratory sequences downloaded from GenBank. We used GARD (Genetic Algorithm for Recombination Detection; Pond et al. 2006) to test for evidence for recombination prior to using tests of selection. We chose the best fitting substitution model using CodonTest (Delport et al. 2010), and then various codon-based tests for selection were implemented. These are tree-based methods that compare the ratio of nonsynonymous changes per nonsynonymous site (*D*_*n*_) to synonymous changes per synonymous site (*D*_*s*_), such that ratios greater than one are indicative of positive selection and ratios less than one indicative of purifying selection. We compared sites predicted to be under positive selection by three codon-based tests for selection described in Pond and Frost (2005): (1) SLAC (Single Likelihood Ancestor Counting), FEL (Fixed Effects Likelihood) and REL (Random Effects Likelihood); (2) MEME (Mixed Effects Models of Evolution), which can detect evidence for diversifying and episodic selection (i.e. a large proportion of positively selected sites); and (3) IFEL (Internal Fixed Effects Likelihood), which investigates population-level selection along internal branches. We also used BSR (Branch-Site REL; Pond et al. 2011) to investigate whether there was evidence for diversifying selection along particular branches of the genealogy.

Tests for deviations from neutrality based on allele frequencies of haplotypes within Wales were implemented using McDonald-Kreitman (McDonald and Kreitman 1991) and HKA tests (Hudson et al. 1987) in DNAsp version 5.10 (Librado and Rozas 2009), using sequences from *X. tropicalis* [as the outgroup because this was the most closely related species available in GenBank (NM_001045794)].

### MHC Class IIb copy number variation

In order to separate sequences into predicted haplotypes according to the three MHC Class IIb loci that have been predicted previously from inbred laboratory-reared *X. laevis* (*DAB*, *DBB* and *DCB*; Kobari et al. 1995), the nucleotide sequence alignment of the exon 2 region was used to reconstruct a genealogy using MEGA 6.0 (Tamura et al. 2013). ModelTest (as implemented in MEGA) was first used to select the best fitting model of evolution, and the maximum likelihood optimality criterion was used to reconstruct trees using the preferred substitution model, with confidence in branching relationships assessed using 1000 bootstrap pseudoreplications.

No MHC Class IIb sequences were available in GenBank from other wild populations of *Xenopus* spp. Thus, haplotype summary statistics (as opposed to population estimates) computed using DNAsp version 5.10 (Librado and Rozas 2009) were used to compare the relative diversity in the three gene copies in Wales to that among published sequences from laboratory colonies, specifically, the number of haplotypes, number of segregating sites and average pairwise genetic diversity based on synonymous (π_s_), nonsynonymous (π_n_) and all sites (π). Sequences were first collapsed into unique haplotypes for each sequence set and separate comparisons made within each for DAB, DBB, DCB and across all loci.

### MHC Class IIb variation compared to other genes

For each gene, summary statistics based on complete genotypes for each individual were computed as for the MHC, but also included Watterson’s *θ* (Watterson 1975). Tajima’s *D* statistic (Tajima 1989) was used to test whether there was evidence for deviation from neutral processes. This statistic compares diversity based on the total number of segregating sites (Watterson’s *θ*) and that based on the average number of mutations between pairs in the sample (π; Nei 1987). Importantly, even in the absence of natural selection, Tajima’s *D* values are also affected by demographic changes (Fay and Wu 1999). An excess of low frequency polymorphisms (e.g. due to population size expansion after a bottleneck, a selective sweep or purifying selection) results in a negative value of *D*, whereas an excess of high frequency polymorphisms (e.g. due to a recent decrease in population size or balancing selection maintaining intermediate frequency variants) results in a positive value of *D*. McDonald-Kreitman and HKA tests were also conducted for each gene, as described for the MHC, using *Xenopus gilli* (accessions HQ221081, HQ221179, EF535964) as the outgroup because it is the most closely related species with sequences available in GenBank.

Since individuals had up to five haplotypes across all MHC Class IIb loci (Table 1), one minus the proportion of homozygous individuals was used as an estimate of heterozygosity that could be compared with the single-copy nuclear genes. In addition, observed (*H*_o_) and expected (*H*_e_) heterozygosity were calculated in Arlequin, version 3.5 (Excoffier and Lischer 2010), assuming complete diploid inheritance (i.e. only two copies of each allele per locus). These values were used to test whether observed heterozygosity deviated from that expected under Hardy-Weinberg Equilibrium (HWE) using Fisher’s exact tests, as implemented in Arlequin.

**Table 1:** Haplotypes (indicated by assigned number) present for each of the three MHC Class IIb loci (DAB, DBB, DCB) in the Welsh population (complete individual names are provided in Additional file: Table S1, for comparison with Tinsley et al. 2012)

Individual	DAB	DBB	DCB
1	3	2	5
2	3,4		5
3	1,3		
4	1		
5	3	2	5
6	3,4		8
7	1,4,7	9	
8	1,3,7	2	
9	1,3	2	
10	1,4	6	8
11	1,3,7		
12	1		
13	3,4	2,6	5
14	1,4		
15	1,4	6	
16	4	6	8
17	4	2,6	
18	3	2	5

Evidence for linkage disequilibrium between patterns of variation at the MHC and the other nuclear genes was investigated using contingency chi-squared analyses, with probability assessed through likelihood ratio tests (as implemented in JMP10.0; SAS Institute Inc. 2012) to assess whether: (1) individuals sharing genotypes at one of the other loci also shared *DAB* genotypes; (2) there was an association between heterozygosity of *DAB* (both in terms of the number of alleles and a binary classification of heterozygosity) and the other nuclear genes; or (3) between the overall number of Class IIb alleles and heterozygosity at the other genes.

To create an individual-based measure of genetic distance that simultaneously accounts for heterozygosity and relative diversity, pairwise distances among alleles found within individuals were calculated for each locus. Significant associations between variation at the MHC and the other nuclear genes were tested using regression analyses, with within-individual distances at the MHC used as the explanatory variable.

The sample sizes were too small to adopt a full general linear model approach to test for sources of variation in genetic diversity in relation to individual data recorded during the mark recapture study (Additional file: Tables S1, S5). However, to specifically test whether there were changes in genetic diversity of heterozygosity over time, linear regression analyses were conducted to determine whether date of birth (either as a continuous trait or a discrete classification of two cohorts, 1993 vs. ≥1999) explained variation at any of the genes (also considered in continuous and discrete classifications).

### Source of the Welsh population

In order to identify the geographic origin of the most closely related mtDNA haplotypes to the Welsh samples, geo-referenced *X. laevis* sequences for the 16S rDNA gene of the mitochondrial genome were downloaded from GenBank and used to create a phylogenetic tree, using MEGA 6.0, as described for the MHC Class IIb genes. These 16S sequences included representatives from many portions of sub-Saharan Africa (Evans et al. 2004) and so could be used to check the source of the released strains. For the single-copy nuclear genes, region of origin was also confirmed based on BLAST alignment to sequences available in GenBank and for two loci (*Prmt6* and *Mogs*) to homologous sequences from *X. laevis* samples collected from over a dozen countries in sub-Saharan Africa (Furman et al. 2015).

## Results

### Selection at the MHC

A total of 14 MHC Class IIb haplotypes were identified among published sequences from laboratory colonies, two of which were shared with the Welsh population. The Welsh population had an additional seven haplotypes, which were similar, but not identical, to the published sequences. Among the Welsh samples, over the 64 codons (192 bp) examined, 40 were variable (comprising 69 variable nucleotide sites), which were distributed in nine haplotypes (GenBank accession numbers KP745469–77). Putative recombinant sequences were identified in five individuals, but these were assumed to be PCR recombinants as opposed to true haplotypes (Additional file: Table S2) and excluded from further analyses. Haplotypes 4 and 7 differed by four synonymous substitutions (all in third base pair positions), and haplotypes 3 and 4 differed from one another by one synonymous and one nonsynonymous substitution. The most divergent haplotypes (1 and 9) differed from one another by 38 nonsynonymous substitutions. Two individuals (11%) were homozygous for haplotype 1; all other individuals had between two and five haplotypes. The number of haplotypes detected was not correlated with the number of clones sequenced (Additional file: Fig. S1 and Table S2).

Aligning these Welsh *X. laevis* sequences with those available in GenBank showed that there were 26 conserved and 38 variable amino acid residues in the sequenced region of the MHC, with 36 of the variable sites in predicted hypervariable regions (HVR) and 16 at predicted residues involved in peptide binding, only one of which is outside an HVR (Fig. 1). There was also a remarkable degree of conservation between *X. laevis* sequences and those from the canid samples described by Marsden et al. (2009). Evidence for positive selection was found at multiple codons using multiple tests for selection, despite the short length of the sequenced region, but these were concentrated at antigen binding sites (see Fig. 1). No strong evidence was found for strong diversifying selection overall, but there was some evidence for episodic selection along particular branches of the genealogy (Additional file: Fig. S4). Consistent with this, haplotype-based tests for selection (McDonald-Kreitman and HKA tests) were not significant.

### MHC Class IIb copy number variation

**Fig. 2:**
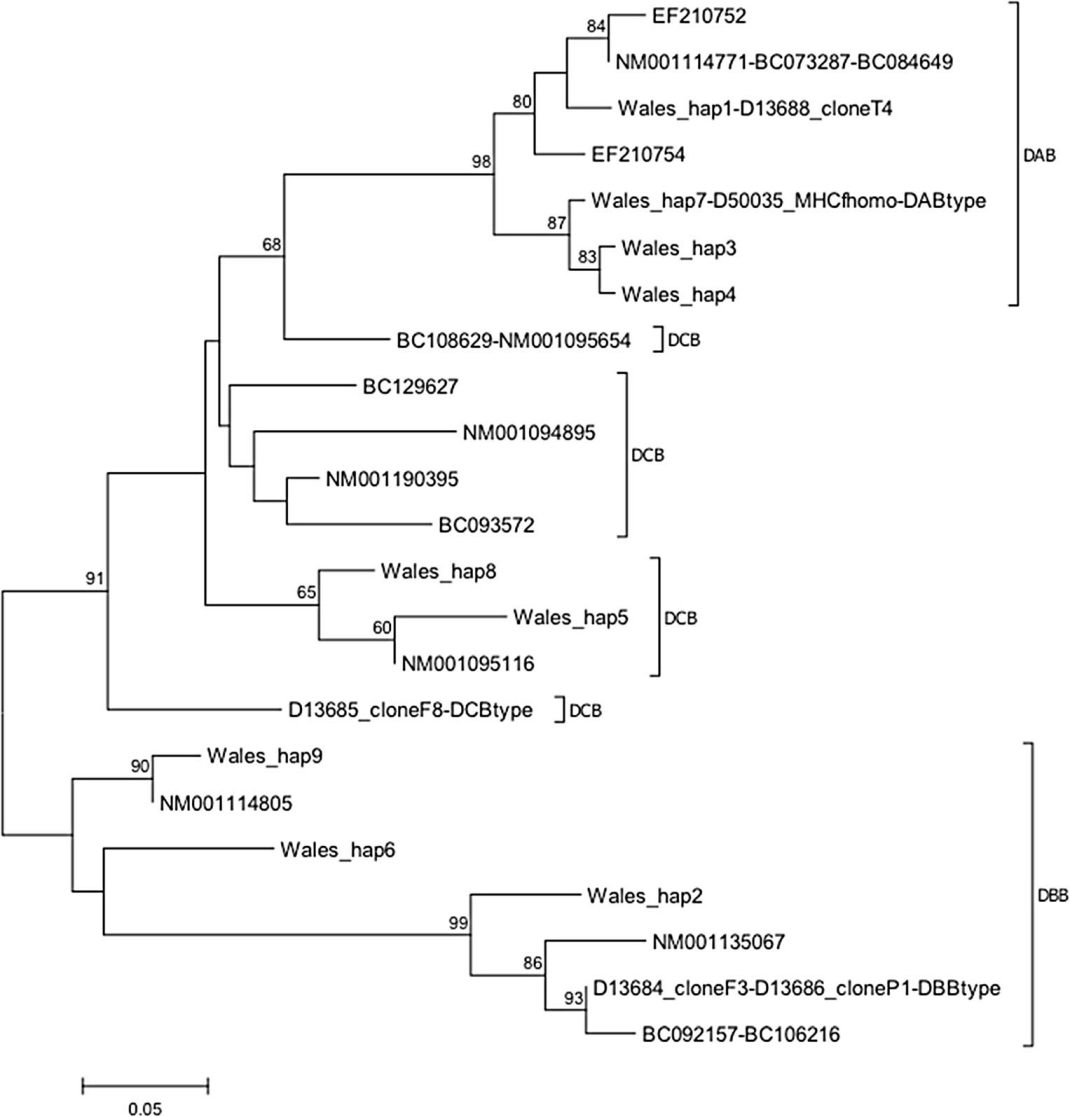
Mid-point rooted maximum likelihood genealogy for Class IIb sequences from Wales and published sequences, indicating the three putative loci described by Kobari et al. (1995); ‘type’ refers to the reference sequence for each locus reported in that paper. Bootstrap support (500 replicates) indicated when greater than 60 %. The Welsh sequences clearly cluster into DAB-like and DBB-like sequence types, but the remaining sequences do not resolve into a clearly distinctive grouping. For ease of comparison, they were designated as DCB. DAB-like genotypes segregated as expected for a single locus, but DCB (designated as Welsh haplotypes 5 and 8) and DBB were predominantly present as homozygotes. At least one sequence designated as DAB was present in all individuals sampled whereas DBB and DCB appeared to show presence/absence variation (PAV) among individuals (see Table 1)

The genealogy of the nine Welsh MHC-based haplotypes and all available sequences from GenBank (Fig. 2) was used to resolve the three Class IIb copies previously proposed (i.e. *DAB*, *DBB* and *DCB*; Kobari et al. 1995), though not all nodes were strongly supported. *DAB* sequences formed a monophyletic group with relatively shallow branch lengths (Additional file: Fig. S3) and included two Welsh haplotypes that were identical to published sequences from laboratory studies: haplotype 1 to clone T4 (Sato et al. (1993) and haplotype 7 to an MHCf homozygote (Kobari et al. (1995). Putative *DBB* sequences also formed a clearly resolved group but with higher among-haplotype divergence. None of these haplotypes was identical to published sequences, but haplotype 2 was most closely related to the *DBB* ‘type’ of Kobari et al. (1995), and haplotype 6 was identical in amino acid sequence to NM00114805 from Klein et al. (2002). Putative *DCB* sequences could not be classified so confidently, but this involved only two haplotypes (Fig. 2). All individuals had at least one of the four *DAB* haplotypes (Additional file: Fig. S4), but presence of the other loci varied by individual (Table 1).

Summary statistics based on unique haplotypes (Table 2) showed that there was a substantial amount of genetic diversity at the MHC Class IIb, both within and between putative loci. Across all three loci, there was a similar level of synonymous diversity for Welsh and laboratory colony samples, but there tended to be lower nonsynonymous diversity among the Welsh samples. Across both sequence sets, there was consistently higher genetic distance between, rather than within, loci, with the largest difference between *DAB* and *DBB* (Additional file: Table S3). There were no substantial differences between π_n_ and π_s_ for any of the gene copies (Table 2), reflecting the overall conservation of polymorphism across the sequence and restriction of nonsynonymous variation to PBS (Fig. 1).

**Table 2:** Comparison of genetic diversity based on unique haplotypes for new samples from Wales, published sequences from laboratory colonies (Lab) and considered across all (All), indicating number of unique haplotypes (Nhap); number of segregating sites (S); average pairwise genetic diversity based on synonymous (π_s_), nonsynonymous (π_n_) and all sites (π)

Locus	Pop	Nhap	S	π_s_	π_n_	π
*DAB*	Wales	4	16	0.073	0.037	0.044
	Lab	5^a^	13	0.012	0.048	0.040
	All	7	16	0.040	0.046	0.045
*DBB*	Wales	3	36	0.120	0.133	0.131
	Lab	4	32	0.062	0.104	0.095
	All	7	40	0.107	0.103	0.107
*DCB*	Wales	2	12	0.000	0.080	0.063
	Lab	7	43	0.057	0.104	0.094
	All	9	43	0.049	0.100	0.089
All loci	Wales	9	64	0.158	0.149	0.151
	Lab	16	65	0.154	0.166	0.162
	All	23	70	0.157	0.165	0.162

^a^Two published haplotypes were identical to those in Wales

### MHC Class IIb variation compared to other genes

The three single-copy nuclear genes were polymorphic (though an order of magnitude less variable compared with the MHC) and showed substantial heterozygosity in the Welsh population (Tables 3 and 4) (GenBank accession numbers KP745478–531). For the housekeeping genes, five polymorphic sites were resolved into five distinct haplotypes for *Prmt6* and three polymorphic sites were resolved into three distinct haplotypes for *Mogs*. Although *Rag2* is a gene whose products have known immunological functions, only three polymorphic sites were found among the four haplotypes resolved. Haplotype diversity and number of haplotypes were comparable to that for *DAB* at *Prmt6* and *Rag2*, but *Mogs* showed lower variation (Table 3). Gene diversity at synonymous, nonsynonymous and all sites showed an order of magnitude higher variation at DAB compared to the other nuclear genes. Heterozygosity at *DAB* was lower than for *Prmt6* and *Rag2* but higher than *Mogs.* Tajima’s *D* was positive for all genes, reflected in the lower diversity based on Watterson’s *θ* than pairwise nucleotide diversity (π), and was significant for *Mogs*, *Rag2* and *DAB*. When considered across all three MHC Class IIb loci, heterozygosity was higher than for any of the other nuclear genes and diversity was up to two orders of magnitude higher; Tajima’s *D* was positive but not significant, which might be expected if multiple loci were included in the same test. No significant deviation from neutrality was detected for any of the genes, using McDonald-Kreitman or HKA tests.

**Table 3:** Comparison of summary statistics at the MHC with two housekeeping loci (*Prmt*6, *Mog*s) and an immune-related gene (*Rag*2), indicating number of individuals sequenced (N); number of sequences compared (Ns); sequence length (L); number of segregating sites (S); number of haplotypes (Nhap); haplotype (gene) diversity; proportion of individuals that were homozygous for each locus; average pairwise genetic diversity based on synonymous (π_s_), nonsynonymous (π_n_) and all sites (π); and genetic diversity based on segregating sites (*θ*) and Tajima’s *D* statistic (significant values are indicated in bold)

Locus	Ns^a^	L	S	Nhap	Hap diversity	Pro. Homo	π_s_	π_n_	π	*θ*	Tajima’s *D*
*Prmt*6	36	642	5	5	0.78	0.17	0.0063	0.0021	0.0031	0.0019	1.65
*Mogs*	36	660	3	3	0.52	0.56	0.0063	0.0010	0.0022	0.0011	**2.37**
*Rag*2	36	1007	3	4	0.68	0.28	0.0044	0.0005	0.0014	0.0007	**2.09**
*DAB*	39	192	16	4	0.73	0.39	0.0479	0.0333	0.0360	0.0200	**2.57**
Class IIb	53	192	64	9	0.87	0.11	0.1426	0.1200	0.1249	0.0991	0.91

Summary statistics are shown for DAB alone and across all of the Class IIb haplotypes identified

^a^Note that the three single-copy nuclear genes behave as if diploid so there are two sequences per individual in the dataset; at the MHC, some individuals had more than two haplotypes and multiple copies, so there were 1–5 sequences per individual for these genes

**Table 4:**
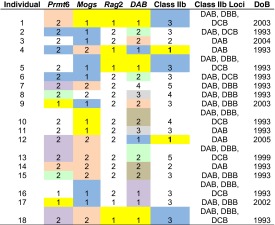
Correspondence between genotypes across loci, showing the number of haplotypes per locus, with the MHC Class IIb indicated in terms of DAB alone, across all loci (Class IIb all) and which Class IIb loci were present; date of birth (DoB) of individuals is also indicated

Colours indicate genotypes shared among individuals within loci (no colour indicates unique genotypes)

Comparing observed (*H*_o_) and expected (*H*_e_) heterozygosity based on haplotype frequencies showed no evidence for excessive inbreeding in the Welsh samples (Additional file: Table S4). At *DAB*, *H*_o_ was less than *H*_e_, but the difference was not significant. Although the number of expected classes was small, DAB, *Prmt6* and *Rag2* segregated according to a model for a single diploid locus, with no deviations from Hardy-Weinberg expectations based on the frequency of each haplotype. Observed heterozygosity was lower than expected at *Mogs*, but the deviation was not significant.

There was little correspondence between the number of haplotypes (either at the MHC overall or *DAB* separately) and heterozygosity across the other nuclear genes or between genotypes at the two sets of loci (Table 4). However, a *χ*^2^ square contingency test revealed a significant association between the number of *DAB* haplotypes and heterozygosity at *Rag2* (Additional file: Fig. S6c; *p* = 0.0016): all *DAB* heterozygotes were also heterozygous at *Rag2* but some of the *DAB* homozygotes were heterozygous at *Rag2*. There was no significant association for the other loci (Additional file: Fig. S6a, b).

There were no significant regressions between birth date (considered as a continuous trait) and the continuous response variables: (1) the number of Class IIb haplotypes, (2) the number of Class IIb loci, (3) the number of *DAB* alleles, (4) within-individual distances for Class IIb overall or *DAB* separately and (5) within-individual distances for any of the single copy nuclear genes (Additional file: Table S5; Table 4). While all slopes were negative, they were extremely shallow and the model explained a very small amount of the variation. There also were no significant differences when birth date was considered by cohort (1993 vs. >1999) for any of these response variables (Additional file: Table S5), nor for the binomial response variables: (1) presence of DBB or DCB; and (2) heterozygosity of any of the genes (Additional file: Table S5).

### Source of the Welsh population

Only one 890-bp mitochondrial 16S haplotype was recovered from the Welsh samples which, by BLAST analysis, was identical to a published sequence from an individual collected from the Western Cape Province of South Africa [accession AY581639, from *X. laevis* isolate Ig 12.3 (Evans et al. 2004); this sample was collected near Cape Town from Lewis Gay Dam, Western Cape Province, South Africa]. This haplotype was also present in the areas of De Doorns and Hoekwil in the southwest Western Cape Province, South Africa (Furman et al. 2015). Except for a segment of a complete mitochondrial genome sequence (HM991335) from an individual collected from Jonkershoek, South Africa (which is ~50 km from Cape Town), all other published sequences from wild-collected individuals from other parts of Africa were more substantially diverged and more distantly related (Additional file: Fig. S5). For the single-copy nuclear genes, the Welsh sequences also were identical to those found in the southwest Western Cape Province of SA (see Additional file: Fig. S2): (1) Garden Route National Park, De Doorns, and Laignsburg, for *Prmt6*; (2) Garden Route National Park, De Doorns, and Laignsburg, Beaufort West, Hoekwil, Niewoudtville and Betty’s Bay for *Mog*s; and (3) Klapmuts, Blackheath and Betty’s Bay for *Rag2* (as well as sequences from individuals obtained from commercial suppliers).

## Discussion

Our data provide the first assessment of MHC variation in a randomly breeding ‘wild’ population of *Xenopus* and suggest that copy number varies among individuals in this closed breeding population. Furthermore, we found that the introduced population of *X. laevis* in Wales has maintained substantial genetic diversity at both immune and housekeeping genes, despite potentially being founded by only a small number of individuals and isolated in a novel habitat for around 50 years.

### Selection at the MHC

Despite the short length of the sequenced region (64 codons), multiple sites of the Class IIb region were found to be under positive selection, most of which were either in predicted HVR regions or at particular sites that have been implicated in peptide binding in humans (Bondinas et al. 2007). There was also evidence for episodic selection along branches, particularly those leading to Welsh haplotypes (Fig. 2). As expected for the MHC, there was substantial divergence among haplotypes when considered over all sequence variants (Fig. 1), despite the large conservation of amino acids that has been noted previously between *Xenopus* and humans (Ohta et al. 2006). For the population-level analyses within Wales, an excess of nonsynonymous diversity indicative of diversifying (positive) selection was restricted to particular codons involved in antigen recognition, with high conservation of amino acids in the rest of the gene, resulting in no significant selection in tests based on average values across the entire gene sequence (i.e. McDonald-Kreitman and HKA tests). This commonly observed pattern for genes coding for molecules with immune function (Chen et al. 2010; Garrigan and Hedrick 2001; Hughes et al. 1993; van Oosterhout et al. 2006) emphasises the importance of using codon-based tests for selection to assess evidence for positive selection, as most genes will include a combination of selected and conserved sites.

### MHC Class IIb copy number variation

Interpreting the meaning of diversity at the MHC is complicated by separation of the Class IIb region into at least three loci (Kobari et al. 1995). Although the Welsh exon 2 sequences fell into three distinctive groups, the lack of resolution of the genealogy meant that sequences could only be assigned completely confidently to the *DAB* locus. This locus was found in all individuals and segregated as a single diploid Mendelian locus. The presence of three haplotypes for some individuals at this locus contradicts findings based on laboratory colonies that both the Class I and Class II regions of the MHC are effectively diploid in tetraploid *Xenopus*, with physical loss of copies and disomic inheritance (Du Pasquier and Blomberg 1982; Kobari et al. 1995; Kobel and Du Pasquier 1986; Sato et al. 1993; Shum et al. 1993). Dodecaploid *X. ruwenzoriensis* have been shown to retain duplicate expression and show polysomic inheritance, suggesting that the locus is retained in duplicate at higher ploidy levels (Sammut et al. 2002). In contrast, for Lake Tana barbs (a group of fish that have undergone recent WGD), gene copy number increases with ploidy for class Ia but not class II genes (Kruiswijk et al. 2004). For the Welsh *X. laevis* samples, the third haplotype in each case (haplotype 7, which is identical to the *DAB* type allele described by Kobari et al. 1995) is presumably functionally equivalent to haplotype 4, from which it differed by only synonymous changes. Thus, this third haplotype could represent retention of a duplicate locus in some individuals or a recent tandem duplication. Haplotypes 3 and 4 differed from each other by only a single amino acid change that was outside of an HVR, but haplotype 1 was highly distinct from the other haplotypes (Additional file: Fig. S4). Therefore, functional diversity may not be as high as suggested by estimates based on nucleotide diversity.

The DBB and DCB loci were not found in all individuals, which suggests that they are ‘expendable’, despite including more highly divergent haplotypes than the *DAB*. We cannot completely rule out an amplification bias against these loci, but all three were amplified in one third of the individuals and this was not related to the number of clones sequenced; in fact, it tended to be individuals with fewer clones screened that showed amplification of all (Additional file: Table S2). It is also possible that there were divergent sequences at these loci that were not amplified. Nevertheless, presence/absence variation among genes coding for resistance phenotypes is common in plants (Bush et al. 2014; Shen et al. 2006) and copy number at the MHC among wild fish species and populations has been found to be extensive, even in diploids (Figueroa et al. 2001; Flajnik and Kasahara 2001; Milinski 2003; Murray et al. 2000; Sato et al. 1997). Eimes et al. (2011) also found that copy number varied *within* a highly bottlenecked population of prairie chickens, emphasising the dynamic nature of immune-related genes. Variation in copy number at the MHC has not previously been reported for *Xenopus* species, but most of the previous research on immunity has been based on inbred laboratory colonies, rather than natural populations (Klein et al. 2002; Kobari et al. 1995; Sato et al. 1993). It is possible that transfer of the Welsh population to the novel environment in the UK resulted in increased genetic drift, a relaxation of selection pressures to combat pathogens present in their habitats, or selection to maintain particular alleles in response to a novel disease agent. Alternatively, if maintenance of a high number of immune variants is costly, as suggested by the lack of retention of duplicate copies in polyploids and the finding of an optimal number of MHC alleles in fish (Forsberg et al. 2007; Milinski 2003; Reusch et al. 2001), relaxation of selection pressures could result in maintenance of a ‘minimal set’ of immune coding genes in the introduced alien population. Since we focused on a short fragment of exon 2, we cannot determine whether whole copies were lost or just this region, which would result in loss of function, as this is where most of the PBR resides (Kobari et al. 1995). Llaurens et al. (2012) suggested that focusing only on the exon containing the PBR could actually underestimate CNV and selection on the MHC since selection and gene conversion events could also occur outside of this region.

In the Welsh population, it is possible that copy number variation was present in the original founders. However, it is intriguing that homozygotes for a single haplotype (i.e. loss of *DBB* and *DCB* and homozygosity at *DAB*) are maintained in some individuals, whereas others retain all three loci. One of the homozygotes was born in the 1993 cohort and one in 2005, but neither showed records of parasites over the mark-recapture period (Additional file: Table S1). Similarly, four of the individuals that had all three Class IIb loci were born in 1993 and the other two were born in 1999 and 2003; four showed parasite presence and two did not, but both of the latter were born in 1993. It is tempting to predict an association with gender (both homozygotes were female, as were five out of six individuals with all three copies), but nearly twice as many females as males were sampled. The sample size is thus too small to draw definitive conclusions, but if copy number is a selectable trait, this could greatly affect the potential for species to adapt to novel environments or tolerate genetic bottlenecks.

### MHC Class IIb variation compared to other genes

Within the Welsh *X. laevis*, there was no evidence for strong linkage disequilibrium across the three single-copy nuclear genes, and each showed slightly different patterns of variation. There was also no evidence for diversifying selection on these genes, in contrast to the MHC. However, there was an association between the number of *DAB* haplotypes and heterozygosity at the other immune-related gene (*Rag2*). Gene diversity was an order of magnitude higher at *DAB*, but haplotype diversity was similar to that at *Rag2*. Although both genes showed significantly positive values of Tajima’s *D* that would be consistent with balancing selection, the observation of similar patterns at the housekeeping genes is more consistent with a signature of a recent demographic bottleneck (Fay and Wu 1999).

Combined with the overall high levels of heterozygosity observed at all loci and lack of deviation from Hardy-Weinberg expectations, the lack of an association between genetic variation at any of the genes compared with date of birth suggests that the population has not suffered from substantial inbreeding as a result of such a founder effect. This could suggest some mechanism for inbreeding avoidance. The MHC has been implicated in odour-based mate choice in a wide range of vertebrates (reviewed by Kamiya et al. 2014; Piertney and Oliver 2006), including humans (Potts et al. 1994; Roberts et al. 2005), other primates (Schwensow et al. 2008; Setchell et al. 2010), birds (Richardson et al. 2005; Zelano and Edwards 2002), fish (Blais et al. 2007; Eizaguirre et al. 2009; Forsberg et al. 2007; Neff et al. 2008; Reusch et al. 2001; Wedekind et al. 2004), salamanders (Bos et al. 2009) and reptiles (Miller et al. 2009). Higher levels of heterozygosity than expected based on neutral markers have also been observed in highly inbred island populations (e.g. killifish, Sato et al. 2002; Channel Island foxes, Aguilar et al. 2004). However, we did not find that the Class IIb region was maintained at a higher level of heterozygosity than the other loci screened, arguing that MHC-driven disassortative mating to avoid inbreeding has not occurred. Since many male anurans provide specific mating calls that females can use to discriminate between potential mates (Gerhardt 1991; Klump and Gerhardt 1987), there is the potential that females could use mating calls to distinguish genetically different mates, but this has not been tested in relation to genome-wide heterozygosity.

### Source of the Welsh population

The identity of haplotypes at both mitochondrial and single-copy nuclear genes with sequences sampled from the southwest Western Cape region of South Africa is consistent with previous suggestions that most introduced populations originated from animals originally collected from this region for scientific use or the pet trade (Lillo et al. 2013; Measey et al. 2012). This view is supported by haplotypes at the MHC class IIb that were identical to those from laboratory colonies, suggesting that founding could have been by a small number of released individuals, which could have already been partly inbred. It is thus even more intriguing that substantial heterozygosity and diversity has been preserved in a closed population for multiple generations. All of the genes used in this study have been predicted to be single copy in *X. laevis*, but segregation studies have not been conducted to determine whether they segregate consistently with complete diploid inheritance. It is possible that flexibility provided by multisomic inheritance could allow maintenance of higher levels of heterozygosity than would be expected for diploid organisms, even if only two copies are retained for each locus. Additional studies of patterns of inheritance would be required to specifically test whether the hybrid polyploid origin of *X. laevis* enables them to maintain higher levels of diversity than true diploids, despite extensive rediploidisation across the genome.

## Conclusions

We have found a substantial amount of genetic diversity and heterozygosity maintained at both immune-related and housekeeping genes in the alien population of *X. laevis* from Wales. This is despite isolation in a novel environment and evidence for a genetic bottleneck. Importantly, our results are consistent with the results from other studies that copy number of MHC genes may be a variable trait within natural populations, which could have important implications for immune repertoire and flexibility in response to pathogens.

## Additional file

Table S1 Details on mark-recapture and records of Welsh samples. Indicated are the sample number-individual code (corresponding to data presented in Tinsley et al 2012), the year of inferred birth (estimated from size at first capture), the number of years of records and the number of times over that period that individuals were captured. All sampled animals were assayed for infection with the monogenean *Protopolystoma xenopodis* as described in Tinsley et al. (2012). (PDF 129 kb)

Table S2 Results of cloning of Class IIb sequences for Welsh samples. Indicated are: individual label; number of clones per individual found for each sequence variant (haplotype) present; number of readable clones sequenced; number of Class IIb sequences (N Class IIb); and the relative frequency of each sequence variant, in terms of numbers of individuals in which it was found (because allelic dosage per individual could not be determined to estimate allele frequencies). (PDF 127 kb)

Table S3 Comparison of genetic distance within (on the diagonal) and between (below the diagonal) the three putative MHC Class IIb loci for all Welsh and laboratory strain haplotypes. (PDF 123 kb)

Table S4 Comparison of observed (*H*_o_) and expected (*H*_e_) heterozygosity in Wales. Calculated by Arlequin, with deviations from Hardy-Weinberg equilibrium (HWE) tested using Fisher’s exact tests (no significant deviations were found); also indicated are the number of heterozygotes and homozygotes for each gene. (PDF 124 kb)

Table S5 Tests for association between birth date of Welsh samples (considered either as two discrete cohorts or a continuous trait) and genetic variation. Comparisons were made with: (1) the number of Class IIb haplotypes; (2) the number of Class IIb loci; (3) the presence of DBB or DCB; (4) the number of DAB alleles; (5) within-individual distances for Class IIb overall or DAB separately; (6) heterozygosity; or (7) within-individual distances for any of the single-copy nuclear genes. For continuous response variables and date of birth considered as a continuous trait, slopes, *r* ^2^and *p* values are indicated (regression analyses); for date of birth divided into early (1993) and late (>1999) cohorts, mean ± standard deviation and *p* values are indicated (*F* ratios from one-way ANOVA). For categorical response variables, *r* ^2^ and *p* values (based on Likelihood Ratio tests) are indicated for date of birth considered as a continuous trait (logistic regression) and as cohorts (contingency chi-square). A General Linear Model revealed no significant effects of parasite presence, gender, cohort or their interactions on the number of Class IIb haplotypes (Full Model, *r* ^2^ = 0.1; *p* = 0.97), but there was a weakly significant effect of gender on the number of Class IIb loci present [Likelihood ratio test, *p* = 0.0415; Effect Size Coefficient (males compared to females) = −5.056], with the 11 females showing more loci on average (2.27) than the seven males (1.71). However, only one male was born after 1993 and it was positive for parasites, so there was no power to separate effects or draw confident conclusions. (PDF 128 kb)

Figure S1 Number of Class IIb haplotypes found in relation to number of clones sequenced. Between 14 and 21 clones produced readable and nonrecombinant sequences per individual. Up to five haplotypes were found with only 15 clones and one of the completely homozygous individuals had sequences from 21 clones, suggesting that lack of finding of Class IIb copies was not limited by number of clones. (PDF 47.5 kb)

Figure S2 Map of the South African Western Cape region, indicating the most closely related sequences to the Welsh samples. **a** Location of the 16S mtDNA haplotype that was identical to that from the Welsh samples; **b**–**e** sampling site for the Bewick et al. (2011) SA sequence set (**b** EA; **c** XSL; **d** KML, **e** RGL), on Betty’s Bay; **f** location of an identical sequence to Welsh *Rag2* haplotype 1 from Klapmuts, which was also found in individuals from EA and Rgl. Also indicated are locations of additional populations included in Furman et al. (2015) that showed sequences with high similarity to the Welsh samples: **g** Garden Route National Park; **h** De Doorns; **i** Laignsburg; **j** Beaufort West; **k** Hoekwill; **l** Niewoudtville. (PDF 674 kb)

Figure S3 *DAB* haplotype network for Welsh samples generated using TCS. Each branch segment (i.e. between nodes) represents one mutation. Relative haplotype frequency is proportional to the size of the *square* or *circle*. Haplotype 3 was inferred as ancestral (*square*) and haplotype 1 was the most divergent. (PDF 25.5 kb)

Figure S4 Mid-point rooted maximum likelihood genealogy for Class IIb sequences from Wales and published sequences. Indicated are the three putative loci described by Kobari et al. (1995); ‘type’ refers to the reference sequence for each locus reported in that paper. Bootstrap support (500 replicates) indicated when greater than 70 %. The Welsh sequences clearly cluster into DAB-like and DBB-like sequence types, but the remaining sequences do not resolve into a clearly distinctive grouping. For ease of comparison, they were designated as DCB. DAB-like genotypes segregated as expected for a single locus but DCB (designated as Welsh haplotypes 5 and 8) and DBB were predominantly present as homozygotes. At least one sequence designated as DAB was present in all individuals sampled whereas DBB and DCB appeared to show presence/absence variation (PAV) among individuals (see Table 1). *Red* indicates nodes or branches found to be under episodic diversifying selection in the branch-site REL analyses performed using datamonkey (at *p* ≤0.05). The length and width of the *bars* reflects relative strength of diversifying selection. Evidence for positive selection was found for many of the branches separating the Welsh sequences from published laboratory sequences. (PDF 61.5 kb)

Figure S5 Maximum likelihood tree of *X. laevis* 16S sequences downloaded from GenBank. The best-fitting model was general time reversible, with rate heterogeneity modelled using a gamma distribution with five rate categories. All sites were included. The tree was rooted with two isolates from *X. gilli*. There was only a single haplotype present in the Welsh population, which was identical to *X. laevis* isolate Ig_3 (AY581639; collected near Cape Town from Lewis Gay Dam, Cape Province, South Africa 34.20˚S 18.4˚E) and a single base pair different from a whole mtDNA genome sequence (HM991335). (PDF 27.8 kb)

Figure S6 Association between the number of *DAB* alleles in Wales and heterozygosity at **a** *Prmt6*; **b** *Mogs*; and **c** *Rag2*. There was a significant association with *Rag2* (all *DAB* heterozygotes were also heterozygous at *Rag2*, but some of the *DAB* homozygotes were heterozygous at *DAB*; *p* = 0.0016 based on a contingency chi-square likelihood ratio test) but not the other loci. There were no significant associations between presence or absence of parasites or numbers of parasites with the number of *DAB* alleles, the number of Class IIb alleles, which Class IIb alleles were present, or presence or absence of *DCB* and *DBB*. There was insufficient power to test for associations with particular *DAB* alleles. (PDF 47.3 kb)
