# Supplementary material for: Copy number variation and genetic diversity of MHC Class IIb alleles in an alien population of Xenopus laevis
Source: Immunogenetics. 2015 Sep 2;67(10):591–603. doi: 10.1007/s00251-015-0860-3 (PMC4572066; doi:10.1007/s00251-015-0860-3)
Supplement: Supplementary file 1 — Details on mark-recapture and records of Welsh samples. Indicated are the sample number-individual code (corresponding to data presented in Tinsley et al 2012), the year of inferred birth (estimated from size at first capture), the number of years of records and the number of times over that period that individuals were captured. All sampled animals were assayed for infection with the monogenean Protopolystoma xenopodis as described in Tinsley et al. (2012). (PDF 129 kb) [file 251_2015_860_MOESM1_ESM.pdf]

Table S1. Details on mark-recapture and records of Welsh samples. Indicated are: the sample number-individual code (corresponding to data presented in Tinsley et al 2012); the year of inferred birth (estimated from size at first capture); the number of years of records; and the number of times over that period that individuals were captured. All sampled animals were assayed for infection with the monogenean *Protopolystoma xenopodis* as described in Tinsley et al. (2012).

Sample-Individual	Birth year	Gender	No. of years of records	No. of captures	No. of times infected[1]
1-plus35	2003	female	4	8	0
2-860	1993	male	3	4	0
3-plus16	2004	male	4	7	2
4-890	1993	female	10	12	0
5-059	1993	male	10	6	1
6-19.96	1993	female	10	16	0
7-x1	1993	female	10	19	7
8-292	1993	male	9	8	0
9-plus33	2003	female	4	5	0
10-988	1993	female	9	6	0
11-926	1993	male	10	11	2
12-fc88	2005	female	6	3	0
13-jx1	1999	female	9	15	0
14-016	1993	male	10	8	0
15-582	1993	male	15	6	0
16-186	1993	female	10	11	0
17-plus18	2002	female	4	8	1
18-x6	1993	female	10	12	1

[1]An infected episode is the number of separate periods of infection (that is episodes of infection separated by periods of non infection), determined from successive capture events.
