# Supplementary material for: Copy number variation and genetic diversity of MHC Class IIb alleles in an alien population of Xenopus laevis
Source: Immunogenetics. 2015 Sep 2;67(10):591–603. doi: 10.1007/s00251-015-0860-3 (PMC4572066; doi:10.1007/s00251-015-0860-3)
Supplement: Supplementary file 2 — Results of cloning of Class IIb sequences for Welsh samples. Indicated are: individual label; number of clones per individual found for each sequence variant (haplotype) present; number of readable clones sequenced; number of Class IIb sequences (N Class IIb); and the relative frequency of each sequence variant, in terms of numbers of individuals in which it was found (because allelic dosage per individual could not be determined to estimate allele frequencies). (PDF 127 kb) [file 251_2015_860_MOESM2_ESM.pdf]

Table S2. Results of cloning of ClassIIb sequences for Welsh samples. Indicated are: individual label; number of clones per individual found for each sequence variant (haplotype) present; number of readable clones sequenced; number of Class IIb sequences (N Class IIb), and the relative frequency of each sequence variant, in terms of numbers of individuals in which it was found (because allelic dosage per individual could not be determined to estimate allele frequencies).

	Haplotype									
	**DAB**[a]	**DBB**	**DAB**	**DAB**	**DCB**	**DBB**	**DAB**	**DCB**	**DBB**	**N<br>clon<br>es**	**N**
**Individual**	**1**	**2**	**3**	**4**	**5**	**6**	**7**	**8**	**9**		**Class IIb**
1-plus-35		3	16		1					20	3
2-860			11	6	1					18	3
3-plus16	2		16							18[b]	2
4-890	18									18	1
5-059		2	11		2					15	3
6-19.96			7	10				1		18[c]	3
7-x1	4			1			13		2	20[d]	4
8-292	6	1	3				5			15	4
9-plus33	3	7	8							18	3
10-988	3			11		1		2		17[e]	4
11-926	6		1				11			18	3
12-fc88	21									21	1
13-jx1		1	4	4	1	5				15	5
14-016	4			16						20	2
15-582	3			13		2				18[f]	3
16-186				15		3		1		19	3
17-plus18		1		15		2				18	3
18-x<br>6		5	8		1					14	3
**Frequency**	**0.56**	**0.39**	**0.56**	**0.50**	**0.28**	**0.28**	**0.17**	**0.17**	**0.06**		

[a] Classification of individual Class IIb loci, according to similarity to sequences described by Kobari et al. (1995)
[b] One additional clone was a recombinant between haplotypes 1 and 3
[c] Two additional clones were recombinants between haplotypes 3 and 4
[d] Two additional clones were recombinants between haplotypes 1 and 7
[e] One additional clone was a recombinant between haplotypes 4 and 8
[f] One additional clone was a recombinant between haplotypes 1 and 4
