# Supplementary material for: Copy number variation and genetic diversity of MHC Class IIb alleles in an alien population of Xenopus laevis
Source: Immunogenetics. 2015 Sep 2;67(10):591–603. doi: 10.1007/s00251-015-0860-3 (PMC4572066; doi:10.1007/s00251-015-0860-3)
Supplement: Supplementary file 3 — Comparison of genetic distance within (on the diagonal) and between (below the diagonal) the three putative MHC Class IIb loci for all Welsh and laboratory strain haplotypes. (PDF 123 kb) [file 251_2015_860_MOESM3_ESM.pdf]

**Table S3. Comparison of genetic distance within (on the diagonal) and between (below the diagonal) the three putative MHC Class IIb loci, for all Welsh and laboratory strain haplotypes.**

| | **DAB** | **DBB** | **DCB** |
|---|---|---|---|
| **DAB** | 0.042 | | |
| **DBB** | 0.331 | 0.101 | |
| **DCB** | 0.164 | 0.217 | 0.128 |
