# Supplementary material for: Copy number variation and genetic diversity of MHC Class IIb alleles in an alien population of Xenopus laevis
Source: Immunogenetics. 2015 Sep 2;67(10):591–603. doi: 10.1007/s00251-015-0860-3 (PMC4572066; doi:10.1007/s00251-015-0860-3)
Supplement: Supplementary file 4 — Comparison of observed (H o) and expected (H e) heterozygosity in Wales. Calculated by Arlequin, with deviations from Hardy-Weinberg equilibrium (HWE) tested using Fisher’s exact tests (no significant deviations were found); also indicated are the number of heterozygotes and homozygotes for each gene. (PDF 124 kb) [file 251_2015_860_MOESM4_ESM.pdf]

Table S4. Comparison of observed (*Ho*) and expected (*He*) heterozygosity in Wales. Calculated by Arlequin, with deviations from Hardy-Weinberg equilibrium (HWE) tested using Fisher's exact tests (no significant deviations were found); also indicated are the number of heterozygotes and homozygotes for each gene.

Gene	Population	*Ho*	*He*	HWE Test	N Hets	N homo
Prmt6	Wales	0.83	0.78	0.911	15	3
Mogs	Wales	0.44	0.52	0.445	8	10
Rag2	Wales	0.72	0.68	0.800	13	5
DAB	Wales	0.61	0.69	0.879	11	7
