# Supplementary material for: Copy number variation and genetic diversity of MHC Class IIb alleles in an alien population of Xenopus laevis
Source: Immunogenetics. 2015 Sep 2;67(10):591–603. doi: 10.1007/s00251-015-0860-3 (PMC4572066; doi:10.1007/s00251-015-0860-3)
Supplement: Supplementary file 5 — Tests for association between birth date of Welsh samples (considered either as two discrete cohorts or a continuous trait) and genetic variation. Comparisons were made with: (1) the number of Class IIb haplotypes; (2) the number of Class IIb loci; (3) the presence of DBB or DCB; (4) the number of DAB alleles; (5) within-individual distances for Class IIb overall or DAB separately; (6) heterozygosity; or (7) within-individual distances for any of the single-copy nuclear genes. For continuous response variables and date of birth considered as a continuous trait, slopes, r 2and p values are indicated (regression analyses); for date of birth divided into early (1993) and late (>1999) cohorts, mean ± standard deviation and p values are indicated (F ratios from one-way ANOVA). For categorical response variables, r 2 and p values (based on Likelihood Ratio tests) are indicated for date of birth considered as a continuous trait (logistic regression) and as cohorts (contingency chi-square). A General Linear Model revealed no significant effects of parasite presence, gender, cohort or their interactions on the number of Class IIb haplotypes (Full Model, r 2 = 0.1; p = 0.97), but there was a weakly significant effect of gender on the number of Class IIb loci present [Likelihood ratio test, p = 0.0415; Effect Size Coefficient (males compared to females) = −5.056], with the 11 females showing more loci on average (2.27) than the seven males (1.71). However, only one male was born after 1993 and it was positive for parasites, so there was no power to separate effects or draw confident conclusions. (PDF 128 kb) [file 251_2015_860_MOESM5_ESM.pdf]

Table S5. Tests for association between birth date of Welsh samples (considered either as two discrete cohorts or a continuous trait) and genetic variation. Comparisons were made with: 1) the number of Class IIb haplotypes; 2) the number of Class IIb loci; 3) the presence of DBB or DCB; 4) the number of DAB alleles; 5) within-individual distances for Class IIb overall or DAB separately; 6) heterozygosity; or 7) within-individual distances for any of the single copy nuclear genes. For continuous response variables and date of birth considered as a continuous trait, slopes, $r^2$and p values are indicated (regression analyses); for date of birth divided into early (1993) and late (>1999) cohorts, mean±standard deviation and p values are indicated (F ratios from one-way ANOVA). For categorical response variables, r^2 and p values (based on Likelihood Ratio tests) are indicated for date of birth considered as a continuous trait (logistic regression) and as cohorts (contingency chi-square). A General Linear Model revealed no significant effects of parasite presence, gender, cohort or their interactions on the number of Class IIb haplotypes (Full Model: r^2 = 0.1; p = 0.97) but there was a weakly significant effect of gender on the number of class IIb loci present (Likelihood ratio test: p = 0.0415; Effect Size Coefficient [males compared to females] = -5.056), with the 11 females showing more loci on average (2.27) than the 7 males (1.71). However, only one male was born after 1993 and it was positive for parasites so there was no power to separate effects or draw confident conclusions.

**Comparison**	**Continuous**			***Cohorts***		
	**r^2**	***p***	***s***	***1993***	**>1999**	**p**
MHC Variation						
N Class IIb haps	0.041	0.42	-	3.00±0.85	2.83±1.33	0.75
N Class IIb loci	0.017	0.61	-	2.08±0.79	2.00±0.89	0.84
N DAB alleles	0.083	0.25	-	1.91±0.79	1.50±0.55	0.27
**Within-individual distance**						
Class IIb	0.011	0.68	-	0.15±0.11	0.13±0.08	0.78
DAB	0.013	0.65	-	0.04±0.03	0.03±0.04	0.69
Prmt6	0.129	0.14	-	0.0036±0.0018	0.0024±0.0022	0.20
Mogs	0.059	0.33	-	0.0020±0.0022	0.0013±0.0020	0.50
Rag2	0.037	0.45	-	0.0017±0.0012	0.0013±0.0014	0.59
**Binomial**	**r^2**	***p***			**r^2**	***p***
Presence of DBB	0.003	0.93			0.005	0.73
Presence of DCB	0.004	0.77			0.00	1.00
DAB Heterozygosity	0.025	0.44			0.02	0.50
Prmt6 Heterozygosity	0.106	0.20			0.10	0.19
Mogs Heterozygosity	0.037	0.34			0.02	0.50
Rag2 Heterozygosity	0.008	0.60			0.007	0.71
