# Supplementary material for: Copy number variation and genetic diversity of MHC Class IIb alleles in an alien population of Xenopus laevis
Source: Immunogenetics. 2015 Sep 2;67(10):591–603. doi: 10.1007/s00251-015-0860-3 (PMC4572066; doi:10.1007/s00251-015-0860-3)
Supplement: Supplementary file 6 — Number of Class IIb haplotypes found in relation to number of clones sequenced. Between 14 and 21 clones produced readable and nonrecombinant sequences per individual. Up to five haplotypes were found with only 15 clones and one of the completely homozygous individuals had sequences from 21 clones, suggesting that lack of finding of Class IIb copies was not limited by number of clones. (PDF 47.5 kb) [file 251_2015_860_MOESM6_ESM.pdf]

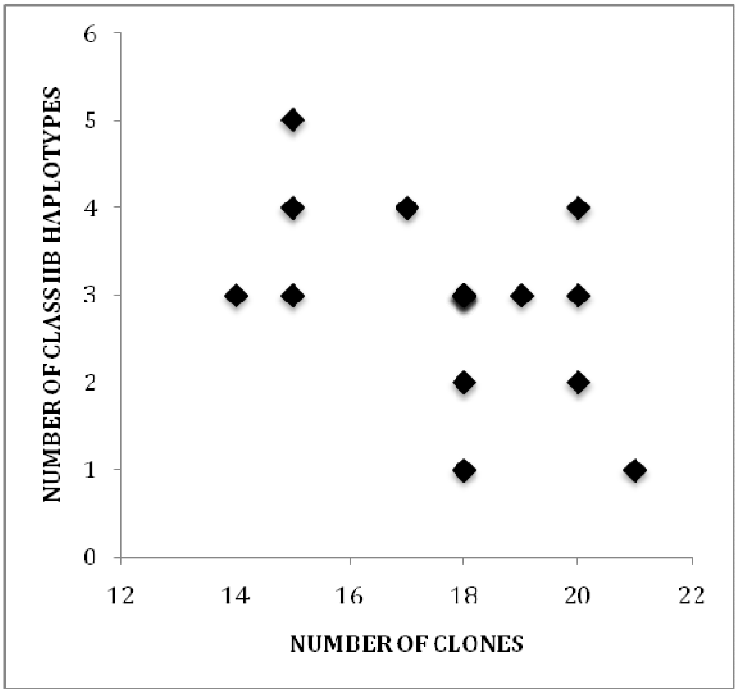

Fig S1.Number of class iib haplotypes found in relation to number of clones sequenced. Between 14 and 21 clones produced readable and nonrecombinant sequences per individual. Up to five haplotypes were found with only 15 clones and one of the completely homozygous individuals had sequences from 21 clones, suggesting that lack of finding of Class IIb copies was not limited by number of clones.
