# Supplementary material for: Copy number variation and genetic diversity of MHC Class IIb alleles in an alien population of Xenopus laevis
Source: Immunogenetics. 2015 Sep 2;67(10):591–603. doi: 10.1007/s00251-015-0860-3 (PMC4572066; doi:10.1007/s00251-015-0860-3)
Supplement: Supplementary file 7 — Map of the South African Western Cape region, indicating the most closely related sequences to the Welsh samples. a Location of the 16S mtDNA haplotype that was identical to that from the Welsh samples; b–e sampling site for the Bewick et al. (2011) SA sequence set (b EA; c XSL; d KML, e RGL), on Betty’s Bay; f location of an identical sequence to Welsh Rag2 haplotype 1 from Klapmuts, which was also found in individuals from EA and Rgl. Also indicated are locations of additional populations included in Furman et al. (2015) that showed sequences with high similarity to the Welsh samples: g Garden Route National Park; h De Doorns; i Laignsburg; j Beaufort West; k Hoekwill; l Niewoudtville. (PDF 674 kb) [file 251_2015_860_MOESM7_ESM.pdf]

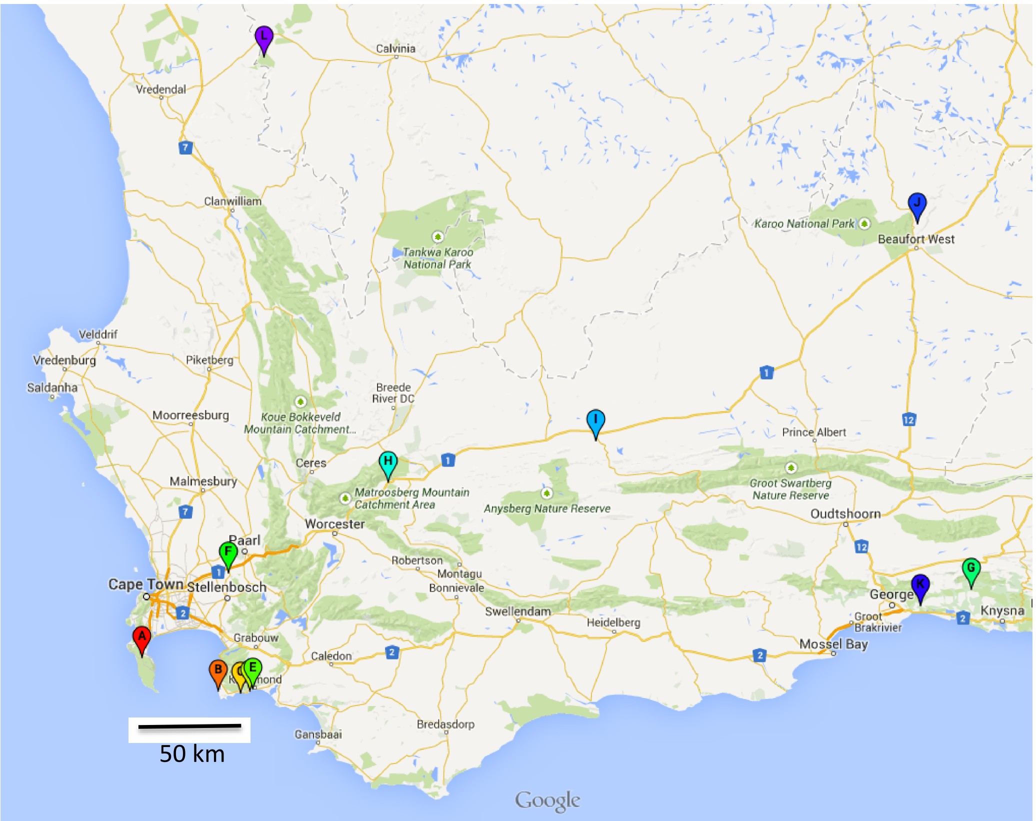

Fig S2.Map of the South African Western Cape region, indicating the most closely related sequences to the Welsh samples. A) location of the 16S mtDNA haplotype that was identical to that from the Welsh samples; B-E) sampling site for the Bewick et al. (2010) SA sequence set (B = EA; C= XSL; D = KML; E = RGL), on Betty’s Bay; F) location of an identical sequence to Welsh *Rag2* haplotype 1 from Klapmuts,which was also found in individuals from EA and Rgl. Also indicated are locations of additional populations included in Furman et al. (2015) that showed sequences with high similarity to the Welsh samples: G = Garden Route National Park; H = De Doorns; I = Laignsburg; J = Beaufort West; K = Hoekwill; L = Niewoudtville.
