# Supplementary material for: Copy number variation and genetic diversity of MHC Class IIb alleles in an alien population of Xenopus laevis
Source: Immunogenetics. 2015 Sep 2;67(10):591–603. doi: 10.1007/s00251-015-0860-3 (PMC4572066; doi:10.1007/s00251-015-0860-3)
Supplement: Supplementary file 8 — DAB haplotype network for Welsh samples generated using TCS. Each branch segment (i.e. between nodes) represents one mutation. Relative haplotype frequency is proportional to the size of the square or circle. Haplotype 3 was inferred as ancestral (square) and haplotype 1 was the most divergent. (PDF 25.5 kb) [file 251_2015_860_MOESM8_ESM.pdf]

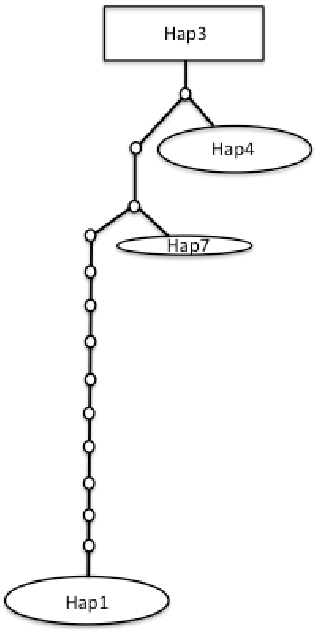

Fig S3.*DAB* haplotype network for Welsh samples generated using TCS. Each branch segment (i.e. between nodes) represents one mutation. Relative haplotype frequency is proportional to the size of the square or circle. Haplotype 3 was inferred as ancestral (square) and haplotype 1 was the most divergent.
