# Supplementary material for: Copy number variation and genetic diversity of MHC Class IIb alleles in an alien population of Xenopus laevis
Source: Immunogenetics. 2015 Sep 2;67(10):591–603. doi: 10.1007/s00251-015-0860-3 (PMC4572066; doi:10.1007/s00251-015-0860-3)
Supplement: Supplementary file 9 — Mid-point rooted maximum likelihood genealogy for Class IIb sequences from Wales and published sequences. Indicated are the three putative loci described by Kobari et al. (1995); ‘type’ refers to the reference sequence for each locus reported in that paper. Bootstrap support (500 replicates) indicated when greater than 70 %. The Welsh sequences clearly cluster into DAB-like and DBB-like sequence types, but the remaining sequences do not resolve into a clearly distinctive grouping. For ease of comparison, they were designated as DCB. DAB-like genotypes segregated as expected for a single locus but DCB (designated as Welsh haplotypes 5 and 8) and DBB were predominantly present as homozygotes. At least one sequence designated as DAB was present in all individuals sampled whereas DBB and DCB appeared to show presence/absence variation (PAV) among individuals (see Table 1). Red indicates nodes or branches found to be under episodic diversifying selection in the branch-site REL analyses performed using datamonkey (at p ≤0.05). The length and width of the bars reflects relative strength of diversifying selection. Evidence for positive selection was found for many of the branches separating the Welsh sequences from published laboratory sequences. (PDF 61.5 kb) [file 251_2015_860_MOESM9_ESM.pdf]

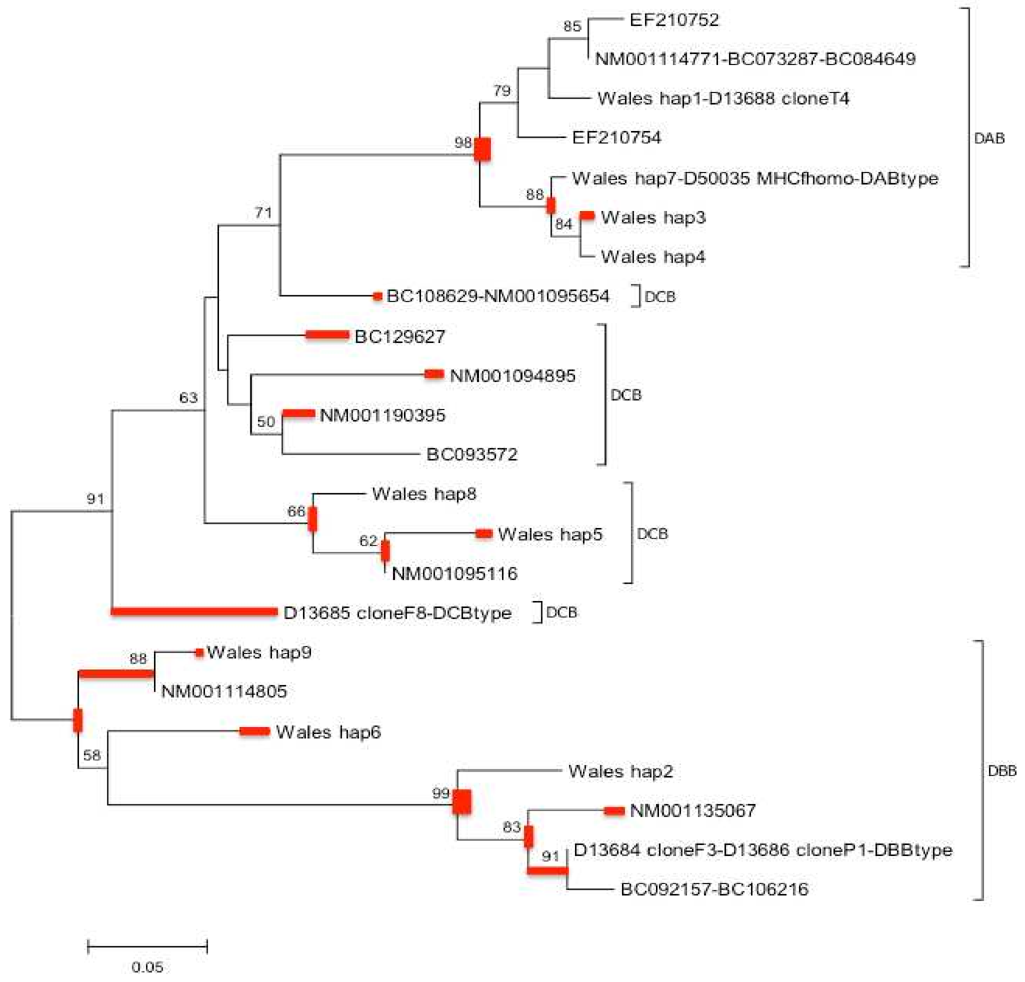

Fig S4.Mid-point rooted maximum likelihood geneology for Class IIb sequences from Wales and published sequences. Indicated are the three putative loci described by Kobari et al. (1995); "type" refers to the reference sequence for each locus reported in that paper. Bootstrap support (500 replicates) indicated when greater than 70%. The Welsh sequences clearly cluster into DAB-like and DBB-like sequence types but the remaining sequences do not resolve into a clearly distinctive grouping. For ease of comparison they were designated as DCB. DAB-like genotypes segregated as expected for a single locus but DCB (designated as Welsh haplotypes 5 and 8) and DBB were predominantly present as homozygotes. At least one sequence designated as DAB was present in all individuals sampled whereas DBB and DCB appeared to show presence/absence variation (PAV) among individuals (see Table 1). Red indicates nodes or branches found to be under episodic diversifying selection in the branch-site REL analyses performed using datamonkey (at $p \leq 0.05$). The length and width of the bars reflects relative strength of diversifying selection. Evidence for positive selection was found for many of the branches separating the Welsh sequences from published laboratory sequences.
