# Supplementary material for: Copy number variation and genetic diversity of MHC Class IIb alleles in an alien population of Xenopus laevis
Source: Immunogenetics. 2015 Sep 2;67(10):591–603. doi: 10.1007/s00251-015-0860-3 (PMC4572066; doi:10.1007/s00251-015-0860-3)
Supplement: Supplementary file 10 — Maximum likelihood tree of X. laevis 16S sequences downloaded from GenBank. The best-fitting model was general time reversible, with rate heterogeneity modelled using a gamma distribution with five rate categories. All sites were included. The tree was rooted with two isolates from X. gilli. There was only a single haplotype present in the Welsh population, which was identical to X. laevis isolate Ig_3 (AY581639; collected near Cape Town from Lewis Gay Dam, Cape Province, South Africa 34.20˚S 18.4˚E) and a single base pair different from a whole mtDNA genome sequence (HM991335). (PDF 27.8 kb) [file 251_2015_860_MOESM10_ESM.pdf]

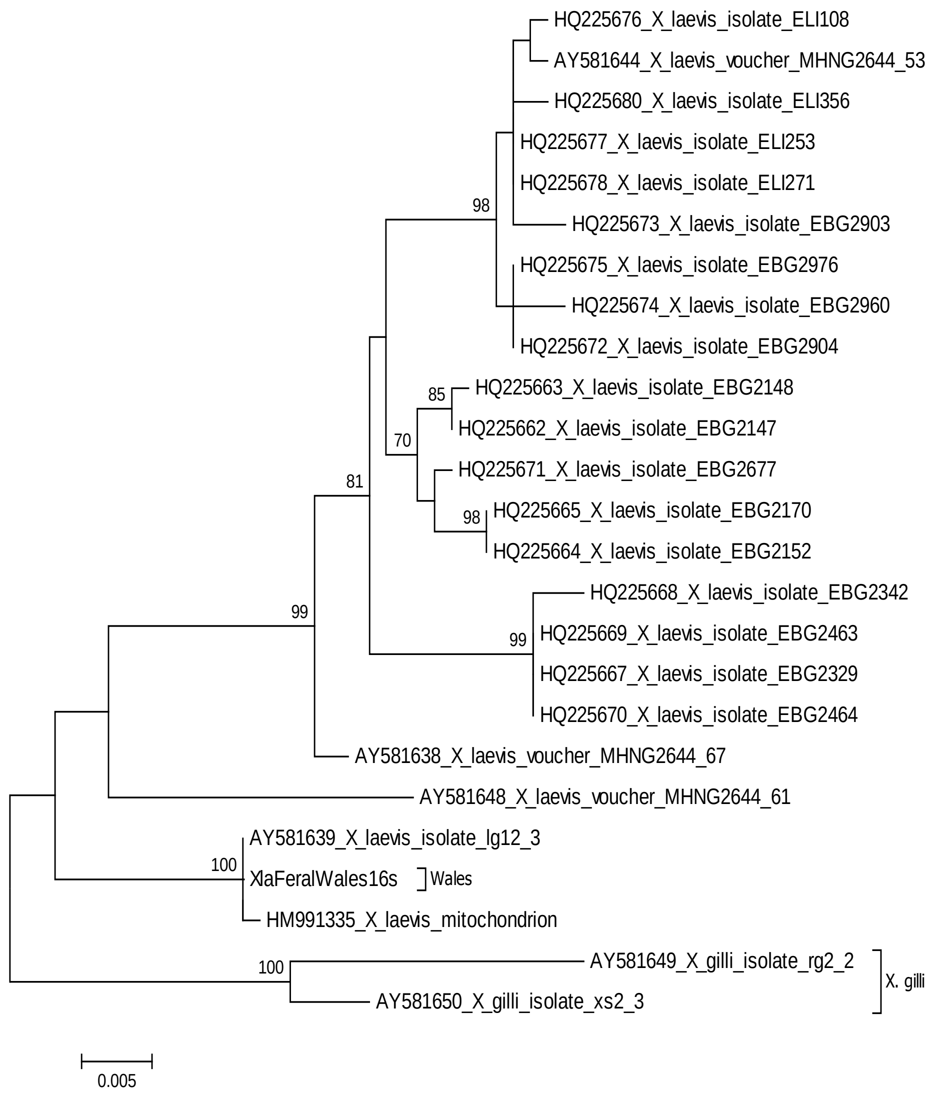

Fig S5.Maximum likelihood tree of *X. laevis* 16S sequences downloaded from Genbank. The best-fitting model was general time reversible, with rate heterogeneity modelled using a gamma distribution with five rate categories. All sites were included. The tree was rooted with two isolates from *X. gilli*. There was only a single haplotype present in the Welsh population, which was identical to *X. laevis* isolate Ig_3 (AY581639; collected near Cape Town from Lewis Gay Dam, Cape Province, South Africa 34.20˚S 18.4˚E) and a single bp different from a whole mtDNA genome sequence (HM991335).
