# Supplementary material for: Copy number variation and genetic diversity of MHC Class IIb alleles in an alien population of Xenopus laevis
Source: Immunogenetics. 2015 Sep 2;67(10):591–603. doi: 10.1007/s00251-015-0860-3 (PMC4572066; doi:10.1007/s00251-015-0860-3)
Supplement: Supplementary file 11 — Association between the number of DAB alleles in Wales and heterozygosity at a Prmt6; b Mogs; and c Rag2. There was a significant association with Rag2 (all DAB heterozygotes were also heterozygous at Rag2, but some of the DAB homozygotes were heterozygous at DAB; p = 0.0016 based on a contingency chi-square likelihood ratio test) but not the other loci. There were no significant associations between presence or absence of parasites or numbers of parasites with the number of DAB alleles, the number of Class IIb alleles, which Class IIb alleles were present, or presence or absence of DCB and DBB. There was insufficient power to test for associations with particular DAB alleles. (PDF 47.3 kb) [file 251_2015_860_MOESM11_ESM.pdf]

a)

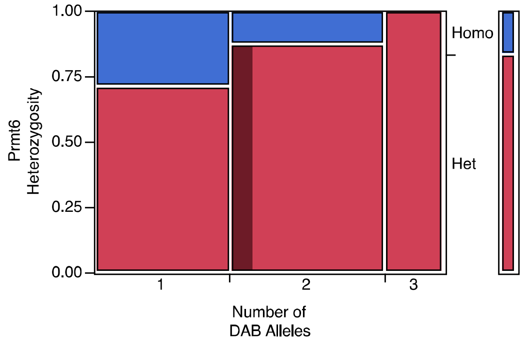

b)

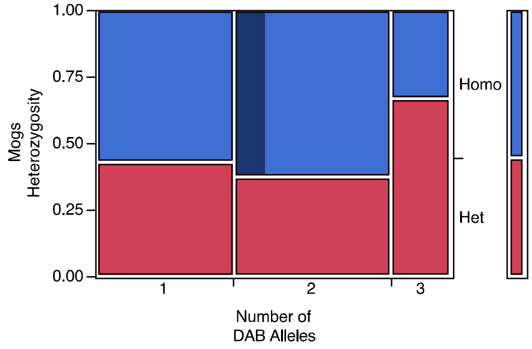

c)

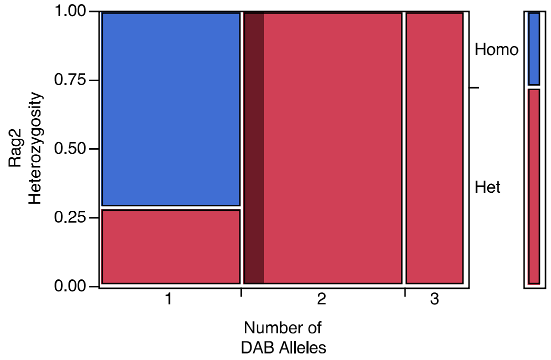

Fig S6.Association between the number of *DAB* alleles in Wales and heterozygosity at: a) *Prmt6*; b) *Mogs*; and c) *Rag2*. There was a significant association with *Rag2* (all *DAB* heterozygotes were also heterozygous at *Rag2* but some of the *DAB* homozygotes were heterozygous at *DAB*; p = 0.0016 based on a contingency chi square likelihood ratio test) but not the other loci. There were no significant associations between presence or absence of parasites or numbers of parasites with: the number of *DAB* alleles; the number of ClassIIb alleles; which ClassIIb alleles were present; or presence or absence of *DCB* and *DBB*. There was insufficient power to test for associations with particular *DAB* alleles.
